# Nanostructure-assisted drought tolerance in olive trees (*Olea europaea* L.): the role of Fe_2_O_3_-graphitic carbon

**DOI:** 10.3389/fpls.2024.1454619

**Published:** 2024-09-04

**Authors:** Rahmatollah Gholami, Narjes Fahadi Hoveizeh, Seyed Morteza Zahedi, Mohsen Padervand, Elmuez A. Dawi, Petronia Carillo

**Affiliations:** ^1^ Crop and Horticultural Science Research Department, Kermanshah Agricultural and Natural Resources Research and Education Center, AREEO, Kermanshah, Iran; ^2^ Department of Horticultural Science, College of Agriculture, Shahid Chamran University of Ahwaz, Ahwaz, Iran; ^3^ Department of Horticultural Science, Faculty of Agriculture, University of Maragheh, Maragheh, Iran; ^4^ Department of Chemistry, Faculty of Science, University of Maragheh, Maragheh, Iran; ^5^ College of Humanities and Sciences, Mathematics and Sciences Department, Ajman University, Ajman, United Arab Emirates; ^6^ Department of Environmental, Biological and Pharmaceutical Sciences and Technologies, University of Campania Luigi Vanvitelli, Caserta, Italy

**Keywords:** carbon nitride, drought stress, nanostructures, olive, osmolytes

## Abstract

Olive trees are known as one of the most iconic crops in the world. Considering the increasing water deficit worldwide, implementing some profitable and empirical strategies can be inevitable upon exposure to drought stress. Therefore, the present study aimed at clarifying the beneficial role of exogenously foliar application of Fe_2_O_3_ modified carbon nitride nanostructures (control, FeSO_4_, C_3_N_4_ and Fe_2_O_3_/g-C_3_N_4_) to “Shengeh” olive cultivars grown at different watering levels (100, 75, and 50% ET) in two experimental years (2022 and 2023) and the pomological attributes, physiological and biochemical changes happening in the treated leaves and fruits were discussed. The results indicated that drought stress caused a significant decline in pomological attributes in this experiment, and treatments could remarkably make up for this damage. Overall, Fe_2_O_3_/g-C_3_N_4_ outperformed as compared FeSO_4_ and C_3_N_4_ alone, which were also efficacious in conferring tolerance to the water deficit stress. Conversely, severe drought stressed-olive fruits showed higher oil content percent in the fresh matter and water use efficiency (WUE) in oil by 30% and 52.5%, respectively, as an average of results of two years, and after Fe_2_O_3_/g-C_3_N_4_, these features in olive plants subjected to severe drought improved by an average of 35% over two years. Ca^2+^ and K^+^ in olive plants under severe drought stress declined by 50% and 83% in 2022 and 46% and 24% in 2023, while Na^+^ increased in the plants exposed to 50%ET stress by 48% and 57% in two successive experimental years respectively. The application of Fe_2_O_3_/g-C_3_N_4_ remarkably improved the contents of Ca^2+^ and K^+^ by 101.5% and 369%, respectively, as an average of two years. Conversely, this beneficial treatment led to a significant decline in Na^+^ levels by 30% in 2022 and 2% in 2023 under stressful conditions. Moreover, it decreased the ‘osmolytes’ content, caused a smaller decline in chlorophyll levels, and resulted in higher relative water content occurring in the treated olive leaves. The reduction of oxidative markers was a result of the increased enzymatic activity after the use of Fe_2_O_3_/g-C_3_N_4_. Therefore, this treatment is a promising strategy to achieve improved resistance in olive plants in the future.

## 1 Introduction

The olive tree (*Olea europaea* L.) is known as one of the most iconic crops not only for its nutritional and economic value but also due to its profound ecological significance (Fraga et al., 2021; Medda et al., 2022; Ferrara et al., 2023). The most important olive-producing countries are Spain, Turkey and Italy, with annual production volumes of 3940070, 2976000 and 2160400 ton respectively; and Iran has up to 114599-ton olive production (Food and Agriculture Organization of the United Nations Statistics, 2022). Phenolic compounds in olive fruit have magnificent nutritional benefits due to their anti-inflammatory, cardio-protective, neuroprotective and anticancer properties (Shi et al., 2017). Despite the olive tree is well suited to the Mediterranean Basin, olive trees are exposed to drought risk, as expected in numerous areas around the world as a consequence of climate change (Meng, 2018).

Drought stress indicates a progressive development in the current era due to the global climate change (Nadeem et al., 2019). Water deficit, through the negative effect on cell division, differentiation as well as enlargement, reduces the plant growth and can degrade chlorophyll (Chl) and suppress photosynthesis (Elkelish et al., 2020). The disruption of Chl causes the reduction of stomatal conductance, photochemical efficiency of PSΙΙ, transpiration rate and photosynthetic electron transport rate (Campos et al., 2019). The plants have an antioxidant system comprising non-enzymatic and enzymatic antioxidants, which systematically control reactive oxygen species (ROS) levels (Talaat et al., 2015). In plant cells, the imbalance between ROS production and scavenging causes oxidative stress during water shortage (Hussain et al., 2018). Excessive ROS-induced drought stress can result in oxidative damage. The severity of damage caused by this secondary stress, most notably H_2_O_2_ (which leads to lipid peroxidation), to the cell membrane is measured by the levels of malondialdehyde (MDA). The plants have evolved the efficacious defense system to cope with oxidative damage including superoxide dismutase (SOD) and catalase (CAT). SOD in the primary step of the defense line. Regulating the O_•_^-2^ status and CAT can control H_2_O_2_ accumulation, converting it to H_2_O (Ubaidillah et al., 2023). However, although high levels of ROS are associated with oxidative stress and cellular damage, moderate levels of ROS, particularly in the early stages of water deficit or during transient exposure to drought, play a crucial role in linking redox status to environmental changes. In fact, a reduction in electron transport efficiency due to a slowdown in the Calvin cycle’s consumption of ATP and NADPH under drought conditions leads to an accumulation of ROS that may elicit retrograde signaling from chloroplast to nucleus for activating genes drought stress response (Li and Kim, 2021). Therefore, the redox reactions of transport chains function as both sensors of environmental conditions and transducers of important signals for acclimation to the environment.

Declined water potential due to drought conditions causes a significant decrease in the uptake of essential minerals like potassium (K^+^) (Ahmad et al., 2018). In severe water-limited conditions, plants suffer from a shortage of nutrients. The lower uptake of these elements from the soil through plant roots and less movement of them in the plant as a consequence of water deficit, lessen the ion content (Shah et al., 2023).

To improve plant resistance to abiotic stresses such as drought, various studies have been carried out, and diverse agronomic strategies have been implemented to achieve stress tolerance in plants exposed to water deficit. The use of some bio-stimulators, such as mineral substances, can protect plants against unwanted environmental conditions (Cui et al., 2017). Iron nanoparticles (Fe-NPs) have been proven able to increase the growth and production of plants as compared with other Fe based fertilizers because of their large surface area to weight ratio (Elanchezhian et al., 2017). The concomitant enhancement in Chl and soluble sugar by the use of Fe-NPs showed that plants use iron to produce photoassimilates (Sreelakshmi et al., 2021). Furthermore, the use of nano Fe_2_O_3_ was more efficacious in the growth of peanut as compared with EDTA-Fe (Rui et al., 2016). In carrots, the uptake of nutrients, the ratio of shoot to root and membrane permeability were improved due to nano-Fe application (Elizabath et al., 2017). It’s valuable to mention that the highest water productivity was obtained with iron Nano-oxide treatment under 50% field capacity (FC) in soybean (Farajollahi et al., 2023).

According to Khodakovskaya et al. (2011), carbon nanomaterials can upregulate several genes involved in stress signalling, such as Les.3648.1.S1 (subtilisin-like endoprotease), Les.3048.1.S1 (DB163 meloidogyne-induced giant cell protein), and LesAffx.64585.1.S1 (threonine deaminase), and promote molecular response, which mimics plant reaction to biotic elicitors such as pathogens or herbivore attacks. In a study, the addition of g-C_3_N_4_ significantly decreased Cd content in the shoot and root of rice (*Oryza sativa* L.), effectively minimizing Cd-induced toxicity (Hao et al., 2021). Also, by exposure of the root and shoot of maize to g-C_3_N_4_ nanosheets, nutrients, Chl content, net photosynthetic rate, electron transfer and carbohydrates increased (Wang et al., 2021).

The use of nanoparticles (NPs) has been demonstrated to improve the tolerance of plants to drought stress much more rapidly than approaches such as genetic improvement alone. In recent years, the application of nano-enabled technology, such as metal-based NPs due to their unique physical and chemical features, has been widely examined in agriculture (Ma et al., 2020). Nanostructures control the release of nutrients according to the nutritional requirements of crops and reduce the application costs and dosages (Zuverza-Mena et al., 2017). Additionally, the stability of nanostructures causes a slower release of nutrients and a beneficial effect on the plant’s growth (Fellet et al., 2021). Li et al. (2020), who used micro-nanostructures, highlighted that this treatment promoted the growth and development of maize plants. Graphitic carbon nitride, as a new two-dimensional conjugated polymer with unique physiochemical traits, is environmentally friendly and cost-effective (Qi et al., 2018). Graphitic carbon can accumulate in the intercellular space of plants and promote root growth by regulating gene expression and enhancement of histone acetylation in the meristem zone in the plant roots (Yan et al., 2016), thus enhancing the uptake of water from drought soils (Zhang et al., 2016).

By considering the mentioned issues above, knowledge about some details about the treatment of carbon nitride remains elusive. The knowledge regarding how this substance modulates the change of growth parameters and biological traits of the plant subjected to drought and the precise combination of this substance should be unraveled or at least improved. Therefore, the present experiment was focused on elucidating the possible influences of exogenous use of Fe_2_O_3_/g-C_3_N_4_ NPs on growth, biochemical and physiological alterations in olive trees, as well as exploring an optimal combination of this material for foliar spraying to mitigate drought stress.

## 2 Material and methods

### 2.1 Fe_2_O_3_/g-C_3_N_4_ nanostructures fabrication

The Fe_2_O_3_ nanoparticles were synthesized by adding the NaBH_4_ solution 1mM drop by drop to a FeCl_3_ solution, whose pH was adjusted to 7 using NaOH solution. The mixture was stirred for one hour, and, in the end, the precipitate was gathered, washed with distilled water repeatedly and then dried at 333 K overnight (1). The C_3_N_4_ powder was prepared by pyrolysis treatment of melamine, 5 g, in a covered crucible at 793 K for 2 h with a heat ramping rate of 10°C/min (2). The pale yellowish product was washed with a diluted nitric acid solution several times to remove unreacted segments. In the end, the equal milligram amounts of Fe_2_O_3_ and C_3_N_4_ powders were dispersed in a beaker containing 150 mL of ethanol using ultrasonication for 60 min. The mixture was then put on a magnetic stirrer at 100°C to evaporate the solvent. The precipitate was washed and then finely powdered to use for practical applications.

### 2.2 Plant material and treatments

This investigation was conducted for two consecutive years (2022 and 2023) at Dallaho Olive Research Station (34° 30ˊ N, 45° 51ˊ E, altitude from sea level: 581 m), Sarpole Zahab, Kermanshah, Iran. This experiment was performed on the ‘Shengeh’ olive cultivar, and agricultural practices such as irrigation, fertilization and weed control of all trees were accomplished uniformly. The spraying treatments including water (control), FeSO_4_, carbon nitride (C_3_N_4_) and Fe_2_O_3_-graphitic carbon nitride nanostructures (Fe_2_O_3_/g-C_3_N_4_), each at a concentration of 0.2 gL^-1^, were applied on 21-years olive trees through full spraying until dripping. These treatments were conducted under three different irrigation regimes: no drought stress as a control (100% evapotranspiration (ET), moderate (75%ET) and severe drought stress (50%ET). The trees were sprayed twice with the aforementioned preparations before flowering and before the fast growth of fruits. Three irrigation regimes were accomplished through drip irrigation, by using the information provided by Gholami et al. (2022). This included the use of daily data from the experimental site, such as maximum and minimum temperature, maximum and minimum and relative humidity, sunshine and wind speed at an elevation of 10 m. Potential evapotranspiration and monthly water needs of olive plants from May to November (without rain) were calculated. Using the Penman-Monteith equation and considering plant coefficients, the amount of water required for irrigating each olive tree every three days was determined separately for the two experimental years. Irrigation was performed every three days, totaling 10 times per month. At the end of the growing season in fall (October 2022 and 2023), fruit, leaf, and shoot samples from the middle part of the trees were collected in the field using sealable bags and transported to the laboratory on ice to measure the morphological and physiological characteristics as described below. Samples to submit to biochemical analyses were immediately shock-frozen in liquid nitrogen (N_2_) and either used immediately for assays or stored at -80°C until use.

### 2.3 Pomological characteristics

Fruit length was measured by a Vernier caliper and fruit diameter was measured via a digital caliper. Fruit weight and pulp fresh weight were measured by an electrical balance. Pulp percent was calculated by this equation:


$$\text{Pulp percent}\left(\%\right)=\frac{\text{Fresh fruit weight}-\text{Fresh pit weight}}{\text{Fresh fruit weight}}\times 100$$

### 2.4 Fruit oil content and fruit yield

The fresh fruits and their pits were dried (70°C) for 48 h. Two grams of fruit pulp was charged to the Soxhlet extractor along with diethyl ether (250 mL) for 5 h then transferred and dried in an oven at 70°C (2 h). The oil content was the difference between the two obtained dried samples (DW). Once the oil content DW is multiplied by the fruit dry matter percentage, the oil content, expressed as FW, was calculated (I.O.O.C, 2002). Fruit yield was calculated by the total harvested fruit content (Kg) in one tree and in one hectare (Sepaskhah et al., 2006).

### 2.5 Water use efficiency

By dividing cumulative fruit production (kg ha^-1^) and total oil production (Lha^-1^) by the cumulative volume of water used (m^3^ha^-1^), the WUE in fruit and oil for each plant were obtained respectively (Sepaskhah et al., 2006).

### 2.6 Enzymatic antioxidants, relative water content and electrolyte leakage

Frozen leaf samples (2 g) were extracted in 1 mL Na-phosphate buffer (50 mM) mixture and then homogenized for 15 min. After adding 0.05 mL of H_2_O_2_ solution, the diaminobenzidine-gelatin, along with horseradish peroxidase, reacted. Peroxidase (POD, EC 1.11.1.7) activity was expressed as unit mg^-1^ protein by measuring the increase in absorbance at 465 nm (Herzog and Fahimi, 1973). Based on the method described by Aebi (1984), frozen leaf tissue was extracted by homogenizing the samples in phosphate buffer (pH 7.0), and the extract was transferred to a cuvette and equilibrated at 25°C in a spectrophotometer. The reaction started by adding a known concentration of hydrogen peroxide (H_2_O_2_) and the decrease in absorbance monitored at 240 nm over time. The decrease in absorbance indicates catalase (CAT, EC 1.11.1.6) activity

The leaves were weighed for their fresh weight (FW). After being rehydrated for 20 h, the leaves were weighed again to obtain the leaf turgid weight (TW). Subsequently, the leaves were dried at 80°C for 48 h and weighed to obtain the dry weight (DW). Ultimately, the relative water content (RWC) was calculated by using the equation below (Gucci et al., 1997):


RWC=100[(FW−DW)/(TW−DW)]

First, the leaves were thoroughly washed with distilled water to remove any superficial contamination. Subsequently, leaf discs were placed in 20 mL of distilled water and shaken at room temperature (150 rpm) for 24 h. The electrolyte leakage (EL) of the solution was measured using an EC-meter (Jenway 4330, USA) and recorded as EL1. Then, the same samples were placed in an autoclave at 121° C for 20 min. After cooling, EL was measured again and recorded as EL2. The final EL was calculated as a percentage of EL1/EL2 (Korkmaz et al., 2007).

### 2.7 Nutritional elements

Olive leaves were dried at 500 ^°^C to obtain ash and then digested (Benton-Jones, 1977). Calcium (Ca^2+^) was determined by atomic absorption spectrometry (Perkin Elmer 3110, USA). K^+^ and Sodium (Na^+^) were measured by a flame photometer (Jenway PSP7, England).

### 2.8 Photosynthetic pigments

One g frozen leaf tissue was dissolved in acetone (100%; 50 mL) and homogenized for 1 min. The obtained homogenate was centrifuged at 2500 rpm (Eppendorf AG 22331, Germany) and the absorbance of the supernatant was recorded at 662 nm and 645 nm by a spectrophotometer (Varian Carry 100 UV, USA) and determined using the equations provided by (Dere et al., 1998):


$$\text{Chl a}=11.75{\text{ A}}_{662}-2.35{\text{ A}}_{645}$$

$$\text{Chl b}=18.61{\text{ A}}_{645}-3.96{\text{ A}}_{662}$$

### 2.9 Osmolytes, total phenolic content and oxidative marker

Leaf samples (0.5 g) were finely ground in 10 mL of 3% sulfosalicylic acid and filtered using Whatman paper. After adding 2 mL of ninhydrin reagent and 2 mL of glacial acetic acid, the sample was placed in a bain-marie at 100 °C. Then, 4 mL of toluene was added, and the proline content was assayed spectrophotometrically at 520 nm, following the protocol of Bates et al. (1973).

Leaf soluble carbohydrates (non-structural carbohydrates, NSC) content was extracted in 80% ethanol from 0.1 g of leaf sample, repeated three times. The extracts were combined to a final volume of 10 mL. 1 mL of extract was mixed with 1 mL of 28% phenol solution and 5 mL of sulfuric acid, then shaken with a vortex mixer. Using the anthrone reagent and the method of Buysse and Merckx (1993) with minor modifications, the absorbance was recorded at 625 nm.

The total phenolic content was quantified according to Singleton and Rossi (1965) using the Folin-Ciocalteu reagent. 250 mg of frozen leaves were extracted in 3 mL of 85% methanol. The extract (300 μL) was combined with Folin-Ciocalteu reagent (10% v), and after 6 minutes, 1.2 mL of 7.5% Na2CO3 was added. The mixture was incubated for 90 minutes at 50 °C, and the absorbance was recorded at 765 nm.

MDA content was measured spectrophotometrically at 532 nm according to Stewart and Bewley (1980). Leaves were initially extracted with 5 mL of distilled water, then 0.5% thiobarbituric acid and 20% trichloroacetic acid solution were added equally. The solution was immediately placed in an ice bath to stop the reaction, and after centrifuging the samples at 10,000 rpm, the absorbance was recorded.

### 2.10 Data analysis

The factorial experiment was conducted according to a complete randomized block with 3 biological replications (every repeat consisted of 24 trees, resulting in 72 trees per treatment). The sources of variance consisted of 4 levels of sprayed treatments and 3 irrigation regimes (4×3 = 12). The obtained experimental data were statistically analyzed by ANOVA using SAS software (v.9.1) and statistical significance was determined by Duncan’s multiple range test at ρ<0.05. The draw of the cluster dendrogram was performed by R.v.3.4.3. The Principal Component Analysis (PCA) was performed using the Minitab 18 statistical software (Minitab LLC, State College, PA, USA).

## 3 Results

### 3.1 Insights into nanomaterial characterization

**Figure 1:**
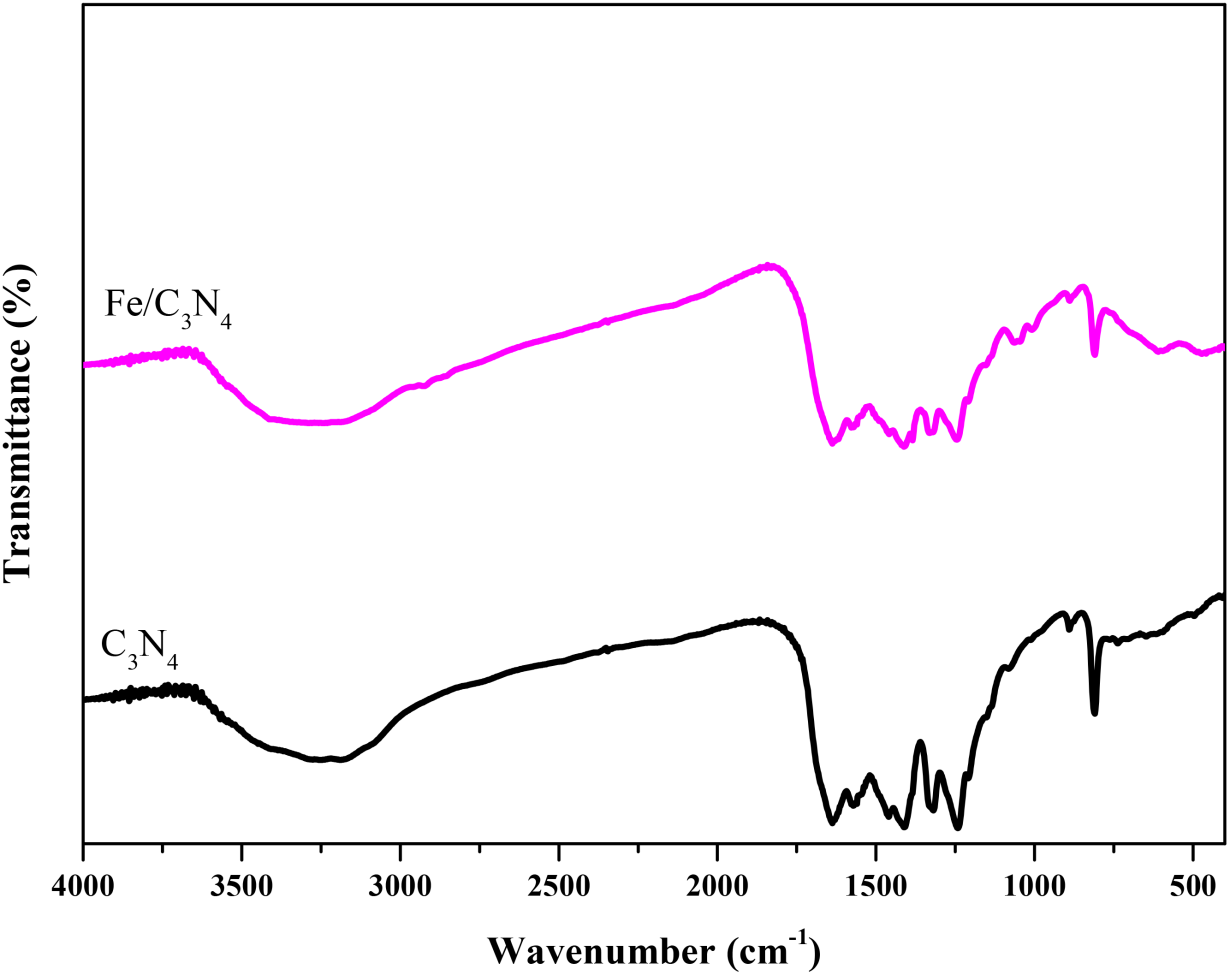
FTIR spectra of C_3_N_4_ and the Fe modified support.

The FTIR spectra of the samples are shown in **Figure 1**. The band at 809 cm^-1^ is assigned to the bending vibration modes of triazine units. Two peaks at 1318 and 1241 cm^-1^ are due to the stretching vibrations of C-NH-C (partial condensation) and C-N(-C)-C (complete condensation) chemical bonds (3). The wide band at 3300 cm^-1^ corresponds to the presence of adsorbed water molecules in the structures. The bands at 1410, 1571 and 1637 cm^-1^ are due to the stretching vibrations of heptazine units (4). The bands at 1200-1560 cm^-1^ are attributed to the stretching vibration modes of CN bonds in the C_3_N_4_ structures (5).

**Figure 2:**
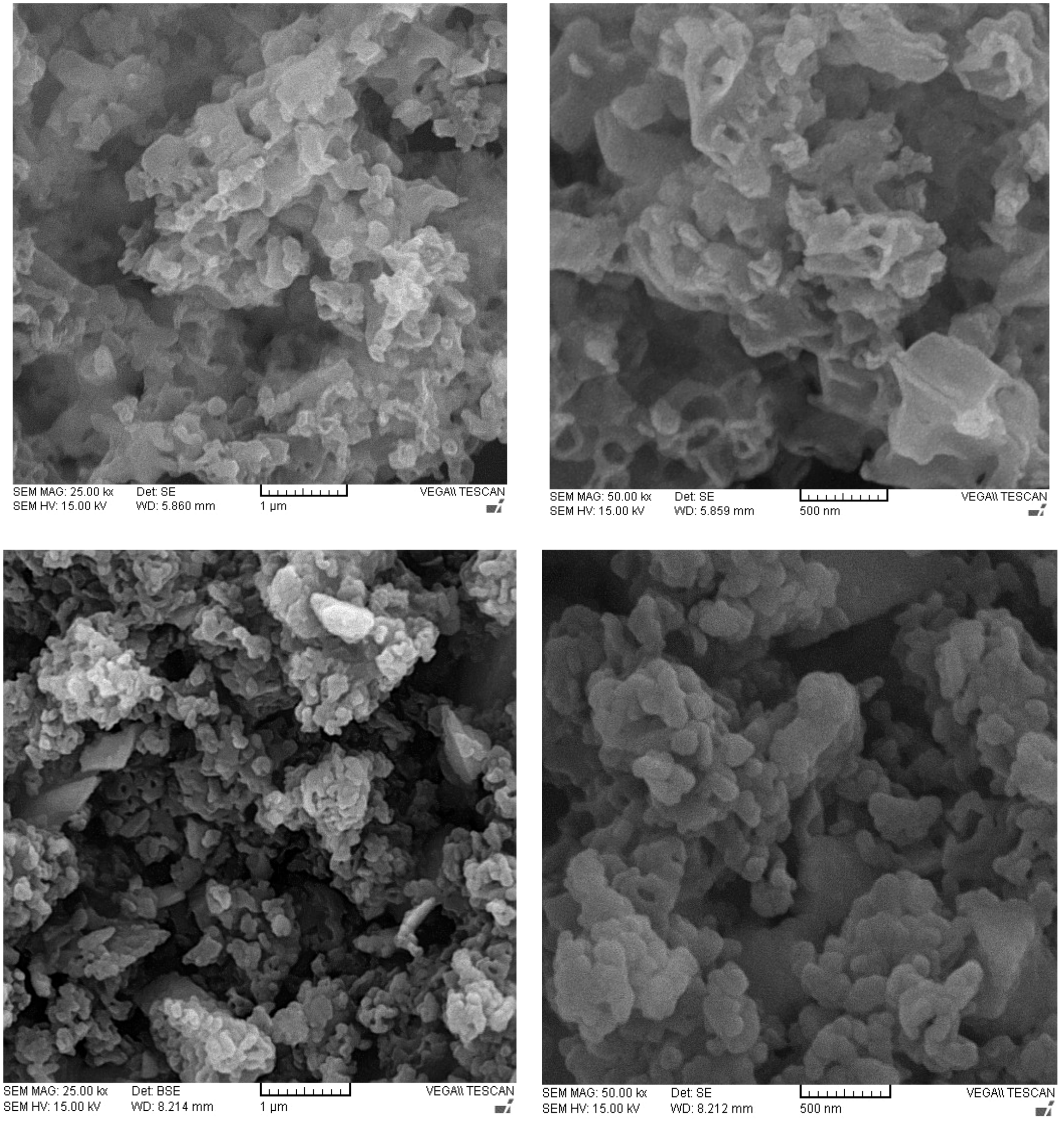
The SEM images of C_3_N_4_ (the two ones up) and Fe/C_3_N_4_ (the two ones down).

The morphology of the structures was explored using SEM images, as shown in **Figure 2**. According to the SEM micrographs of C_3_N_4_, the polymeric structure indicates a unique morphology with smooth porosity, making it a suitable support for carrying extra chemical agents. The thin layers of C_3_N_4_ construction arise from the pyrolysis treatment of the precursors, which is typical for this category of semiconducting materials. With the addition of Fe_2_O_3_ to the surface, as it is obvious, the morphology was clearly changed, and the extra coagulation-like species appeared in the micrographs due to the Fe_2_O_3_ formation on the surface. Such alterations well demonstrate the modification of C_3_N_4_ surface with hematite nanoparticles, generating the Fe_2_O_3_/g-C_3_N_4_ composite structure.

**Figure 3:**
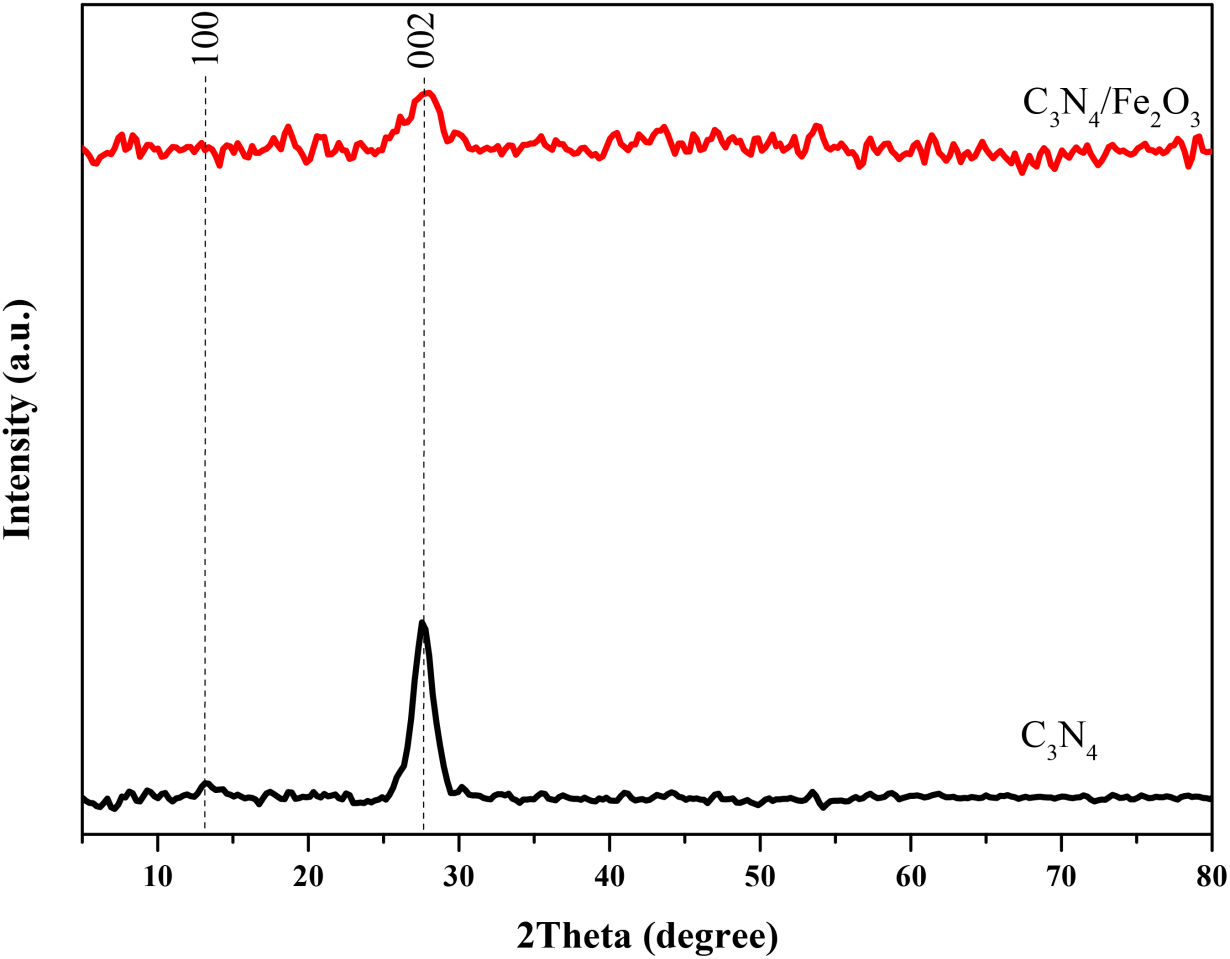
XRD patterns of the Fe_2_O_3_/g-C_3_N_4_.

To explore the crystalline phases of the nanostructures, XRD analysis was performed, and the obtained patterns are shown in **Figure 3**. As it can be seen, two characteristic peaks at 2theta degrees of 13.1 and 27.7 are evident in both patterns. These peaks correspond to the crystalline planes of (100) and (002) in C_3_N_4_ structure, respectively (JCPDS 87-1526). Their intensity significantly decreased with the addition of hematite to the C_3_N_4_ structure, implying that Fe_2_O_3_ particles surrounded graphitic C_3_N_4_ layers. The interference of iron species with the copper K-α x-ray energy used to record the XRD patterns likely caused the appearance of more noises in the Fe_2_O_3_/g-C_3_N_4_ pattern.

### 3.2 Fe_2_O_3_/g-C_3_N_4_ treatment significantly influences pomological traits

**Figure 4:**
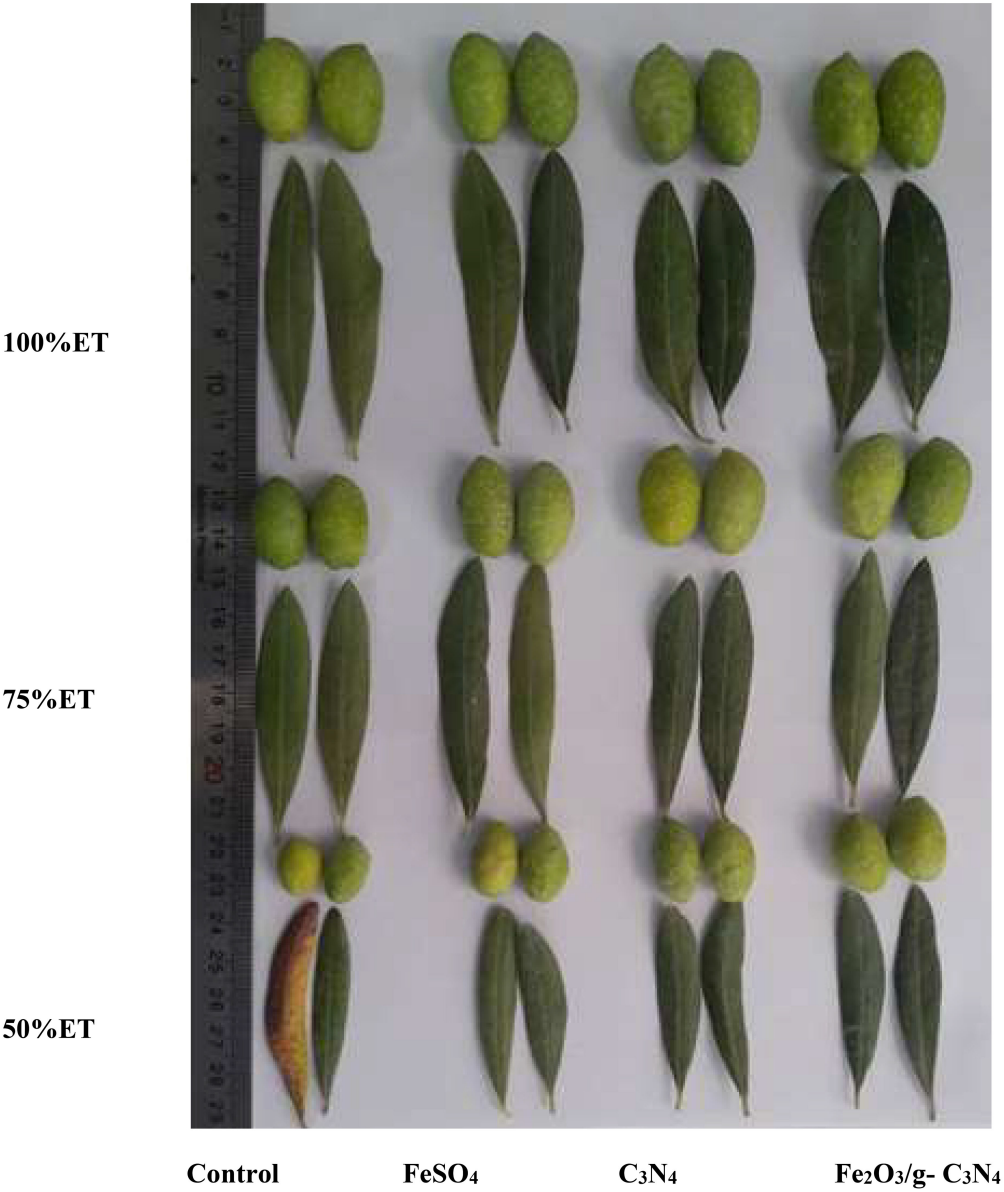
The impact of foliar spraying (from left to right, control, FeSO_4_, C_3_N_4_ and Fe_2_O_3_/g-C_3_N_4_ respectively) of olive (cv. Shengeh) on the appearance as well as dimensions of leaves and fruits under 3 different drought stress levels (from above to bottom, 100%ET or normal condition, 75%ET or moderate drought stress and 50%ET or severe drought stress, respectively).

The changed pomological characteristics as affected by different spraying treatments and irrigation regimes are presented in **Tables 1**, **2**, and the appearance and size of examined olive fruits and leaves are represented in **Figure 4**. Pomological characteristics showed significant delay in a severity-dependent manner under severe drought stress [50% ET] compared to normal conditions (100% ET), with reduction of 50, 9 and 20% in fruit weight, fruit length and fruit diameter, respectively, in 2022. Meanwhile, this reduction of pomological characteristics was observed again in the second experimental year, with decreases of 43, 17 and 26% in fruit weight, fruit length and fruit diameter, respectively, in 2023 (**Table 1**). Under 50%ET, Fe_2_O_3_/g-C_3_N_4_ improved pomological parameters by 80, 25 and 50% compared to no treated control in fruit weight, fruit length and fruit diameter, respectively, in 2022, and by 60, 24 and 24%, respectively, in 2023.

**Table 1:** The effect of spraying treatment, irrigation level and their interactions on fruit weight, fruit length and fruit diameter in *Olea europaea* var. Shengeh.

Source of variance	Fruit weight (g)		Fruit length (cm)		Fruit diameter (cm)	
	2022	2023	2022	2023	2022	2023
Spraying treatment						
WT	2.18±0.61 c	3.02±0.72 d	1.78±0.12 c	2.05±0.19 c	1.06±0.11 d	1.44±0.18 c
FeSO_4_	2.68±0.60 b	3.72±0.64 b	2.04±0.12 b	2.29±0.15 b	1.36±0.09 b	1.53±0.13 b
C_3_N_4_	2.58±0.64 b	3.44±0.66 c	1.99±0.12 b	2.24±0.11 b	1.20±0.12 c	1.53±0.12 b
Fe_2_O_3_/g-C_3_N_4_	3.50±0.73 a	3.08±0.72 a	2.29±0.21 a	2.43±0.18 a	1.50±0.09 a	1.60±0.11 a
Irrigation level						
100%ET	3.42±0.49 a	4.41±0.57 a	2.14±0.24 a	2.41±0.16 a	1.38±0.18 a	1.69±0.07 a
75%ET	2.83±0.56 b	3.40±0.28 b	2.02±0.20 b	2.21±0.16 b	1.28±0.17 b	1.50±0.07 b
50%ET	1.95±0.55 c	2.89±0.50 c	1.90±0.18 c	2.13±0.20 b	1.18±0.18 c	1.39±0.11 c
Spraying treatment×Irrigation level						
WT×100%ET	2.86±0.04	3.77±0.42	1.87±0.09	2.20±0.08 de	1.17±0.09	1.64±0.07 bc
WT×75%ET	2.26±0.04	3.16±0.17	1.78±0.13	2.13±0.09 e	1.07±0.03	1.47±0.05 e
WT×50%ET	1.44±0.30	2.14±0.07	1.70±0.07	1.83±0.04 f	0.94±0.03	1.21±0.25 f
FeSO4×100%ET	3.39±0.14	4.59±0.23	2.18±0.09	2.45±0.04 b	1.45±0.07	1.71±0.05 ab
FeSO4×75%ET	2.69±0.01	3.43±0.05	2.00±0.05	2.17±0.17 e	1.37±0.05	1.47±0.05 e
FeSO4×50%ET	1.97±0.20	3.15±0.08	1.94±0.07	2.26±0.02 cde	1.27±0.04	1.42±0.03 e
C_3_N_4_×100%ET	3.26±0.13	4.27±0.42	2.04±0.09	2.37±0.05 bcd	1.31±0.08	1.68±0.06 ab
C_3_N_4_×75%ET	2.65±0.28	3.23±0.14	2.07±0.05	2.17±0.05 e	1.20±0.08	1.50±0.08 de
C_3_N_4_×50%ET	1.83±0.32	2.83±0.05	1.85±0.02	2.20±0.08 de	1.10±0.08	1.42±0.03 e
Fe_2_O_3_/g-C_3_N_4_×100%ET	4.17±0.09	5.03±0.17	2.50±0.01	2.63±0.05 a	1.61±0.04	1.75±0.04 a
Fe_2_O_3_/g-C_3_N_4_×75%ET	3.73±0.05	3.77±0.20	2.23±0.17	2.40±0.14 bc	1.48±0.02	1.57±0.05 cd
Fe_2_O_3_/g-C_3_N_4_×50%ET	2.60±0.53	3.43±0.29	2.13±0.17	2.27±0.09 bcde	1.41±0.04	1.50±0.00 de
Significance						
Spraying treatment	**	**	**	**	**	**
Irrigation level	**	**	**	**	**	**
Spraying treatment×Irrigation level	ns	ns	ns	*	ns	**

ns, *, ** non-significant or significant at p ≤ 0.05 and 0.01, respectively. Values represent means ± standard deviation of three independent replications (n = 3). Different letters within the same column indicate significant differences at p ≤ 0.05 among the treatments, according to Duncan’s multiple range test. The abbreviation of features name: C_3_N_4_, carbon nitride; Fe_2_O_3_/g-C_3_N_4_, Fe_2_O_3_-graphitic carbon nitride nanostructure; 100%ET, normal condition; 75%ET, moderate drought stress; 50%ET, severe drought stress; WT, water (Control).

**Table 2:** The effect of spraying treatment, irrigation level and their interactions on pulp fresh weight and pulp percent in *Olea europaea* var. Shengeh.

Source of variance	Pulp fresh weight (g)		Pulp percent (%)	
	2022	2023	2022	2023
Spraying treatment				
WT	1.68±0.57 c	2.37±0.57 c	77.56±4.72	78.24±4.69
FeSO_4_	2.04±0.51 b	2.85±0.59 b	75.46±3.66	76.13±2.84
C_3_N_4_	1.97±0.55 bc	2.56±0.59 c	75.59±3.77	75.55±3.63
Fe_2_O_3_/g-C_3_N_4_	2.83±0.65 a	3.18±0.63 a	80.50±3.36	77.81±3.58
Irrigation level				
100%ET	2.72±0.44 a	3.44±0.55 a	78.71±3.06	78.95±3.23
75%ET	2.20±0.51 b	2.58±0.27 b	77.49±4.43	75.97±3.75
50%ET	1.47±0.54 c	2.19±0.41 c	75.63±4.95	75.87±3.90
Spraying treatment×Irrigation level				
WT×100%ET	2.27±0.11	2.96±0.23	77.43±3.17	78.88±2.66
WT×75%ET	1.76±0.13	2.49±0.14	77.93±6.33	78.77±3.53
WT×50%ET	1.01±0.38	1.66±0.19	77.31±4.04	77.07±6.66
FeSO4×100%ET	2.64±0.19	3.62±0.32	77.79±2.67	78.85±3.36
FeSO4×75%ET	2.01±0.08	2.59±0.03	74.64±3.02	75.35±1.04
FeSO4×50%ET	1.46±0.22	2.34±0.05	73.95±3.93	74.18±0.12
C_3_N_4_×100%ET	2.55±0.19	3.21±0.58	78.11±2.67	79.61±1.42
C_3_N_4_×75%ET	2.02±0.22	2.36±0.16	76.24±0.77	73.07±3.26
C_3_N_4_×50%ET	1.33±0.28	2.10±0.07	72.41±4.23	73.98±1.34
Fe_2_O_3_/g-C_3_N_4_×100%ET	3.40±0.12	3.96±0.36	81.52±1.56	78.46±4.54
Fe_2_O_3_/g-C_3_N_4_×75%ET	3.03±0.11	2.90±0.30	81.14±2.35	76.70±3.84
Fe_2_O_3_/g-C_3_N_4_×50%ET	2.07±0.55	2.69±0.26	78.85±4.67	78.27±1.04
Significance				
Spraying treatment	**	**	ns	ns
Irrigation level	**	**	ns	ns
Spraying treatment×Irrigation level	ns	ns	ns	ns

ns, *, ** non-significant or significant at p ≤ 0.05 and 0.01, respectively. Values represent means ± standard deviation of three independent replications (n = 3). Different letters within the same column indicate significant differences at p ≤ 0.05 among the treatments, according to Duncan’s multiple range test. The abbreviation of features name: C_3_N_4_, carbon nitride; Fe_2_O_3_/g-C_3_N_4_, Fe_2_O_3_-graphitic carbon nitride nanostructure; 100%ET, normal condition; 75%ET, moderate drought stress; 50%ET, severe drought stress; WT, water (Control).

As can be seen in **Table 2**, pulp fresh weight decreased by 55% in 2022 and 44% in 2023, under 50%ET compared to 100%ET. According to **Table 2**, irrespective of treatments, drought stress could decrease pulp percent without a significant effect, by 0.15% in 2022 and 2% in 2023 compared to normal conditions.

Although, in some pomological characteristics, which are represented in **Tables 1**, **2**, there was no significant difference between Fe_2_O_3_/g-C_3_N_4_treatment and FeSO_4_ and C_3_N_4_, the application of Fe_2_O_3_/g-C_3_N_4_ significantly improved pomological features under drought stress. In Fe_2_O_3_/g-C_3_N_4_ treated plants, pulp fresh weight increased by 100% in 2022 compared to non-sprayed olive plants under severe drought stress. In addition, under severe drought, pulp percent improved by 2% in 2022 and by 1% in 2023 after treatment with Fe_2_O_3_/g-C_3_N_4_ compared with respective non-treated controls (**Table 2**).

### 3.3 Olive oil content and yield increase under drought with Fe_2_O_3_/g-C_3_N_4_ treatment

Severe drought stressed-olive plants under 50%ET showed higher contents of oil percent in dry matter and oil percent in fresh matter, by 4 and 30% in 2022, and 4 and 29% in 2023, respectively, compared to 100%ET treatments (**Table 3**). In the same condition, the Fe_2_O_3_/g-C_3_N_4_ treatment further increased the oil percent in dry matter by 34% and 37%, and the oil percent in fresh matter by 58% and 12% in 2022 and 2023, respectively (**Table 3**). It is valuable to note that the interaction effect of treatment and irrigation level was not significant in the majority of features in this study.

**Table 3:** The effect of spraying treatment, irrigation level and their interactions on oil percent in dry matter and oil percent in fresh matter in *Olea europaea* var. Shengeh.

Source of variance	Oil percent in dry matter (%)		Oil percent in fresh matter (%)	
	2022	2023	2022	2023
Spraying treatment				
WT	23.95±0.73 d	24.89±2.02 c	5.90±0.87 c	9.05±1.25 a
FeSO_4_	26.76±1.29 c	32.57±3.06 a	7.88±1.24 b	9.55±1.82 a
C_3_N_4_	30.17±2.73 b	27.63±1.50 b	8.51±1.56 b	7.73±1.56 b
Fe_2_O_3_/g-C_3_N_4_	32.39±3.92 a	33.40±2.93 a	9.93±1.78 a	8.99±1.90 a
Irrigation level				
100%ET	27.31±3.74	28.51±4.47 b	6.66±1.46 b	7.41±0.97 c
75%ET	29.33±4.27	30.75±3.73 a	8.53±2.04 a	8.32±1.01 b
50%ET	28.31±3.95	29.61±4.34 ab	8.96±1.71 a	10.77±1.24 a
Spraying treatment×Irrigation level				
WT×100%ET	23.13±0.29	23.33±0.47	5.02±0.28	7.83±0.20
WT×75%ET	24.67±0.47	27.00±2.16	6.17±0.77	9.24±0.79
WT×50%ET	24.07±0.31	24.33±0.47	6.51±0.60	10.08±1.19
FeSO4×100%ET	26.12±0.81	31.74±3.45	6.30±0.13	8.19±0.90
FeSO4×75%ET	27.33±0.94	32.87±2.60	8.21±0.36	8.67±0.70
FeSO4×50%ET	26.83±1.65	33.12±2.91	9.13±0.57	11.80±0.97
C_3_N_4_×100%ET	29.00±2.45	26.63±1.21	6.69±0.38	6.28±0.32
C_3_N_4_×75%ET	31.50±2.68	28.67±1.25	8.95±0.80	7.08±0.31
C_3_N_4_×50%ET	30.00±2.45	27.60±1.28	9.89±1.05	9.84±0.37
Fe_2_O_3_/g-C_3_N_4_×100%ET	31.00±3.71	32.33±3.30	8.65±1.22	7.34±0.88
Fe_2_O_3_/g-C_3_N_4_×75%ET	33.83±3.72	34.47±2.46	10.82±2.05	8.27±0.59
Fe_2_O_3_/g-C_3_N_4_×50%ET	32.33±3.80	33.40±2.57	10.32±1.08	11.35±0.95
Significance				
Spraying treatment	**	**	**	**
Irrigation level	ns	*	**	**
Spraying treatment×Irrigation level	ns	ns	ns	ns

ns, *, ** non-significant or significant at p ≤ 0.05 and 0.01, respectively. Values represent means ± standard deviation of three independent replications (n = 3). Different letters within the same column indicate significant differences at p ≤ 0.05 among the treatments, according to Duncan’s multiple range test. The abbreviation of features name: C_3_N_4_, carbon nitride; Fe_2_O_3_/g-C_3_N_4_, Fe_2_O_3_-graphitic carbon nitride nanostructure; 100%ET, normal condition; 75%ET, moderate drought stress; 50%ET, severe drought stress; WT, water (Control).

As shown in **Table 4**, irrespective of the spraying treatments, olive plants exposed to severe drought stress underwent a reduction of fruit yield per tree and per hectare by 49% in 2022 and 31% in 2023 in comparison with the non-stressed condition. Moreover, olive plants treated with Fe_2_O_3_/g-C_3_N_4_ displayed an increase in both fruit yield per tree and per hectare by 11% and 48% in successive years.

**Table 4:** The effect of spraying treatment, irrigation level and their interactions on fruit yield in tree and fruit yield in hectare in *Olea europaea* var. Shengeh.

Source of variance	Fruit yield in tree (Kg.tree^-1^)		Fruit yield in hectare (Kg.ha^-1^)	
	2022	2023	2022	2023
Spraying treatment				
WT	20.17±5.46 b	21.43±3.29 c	5601.70±1515.76 b	5952.70±915.34 c
FeSO_4_	22.44±5.99 a	23.70±2.69 b	6234.40±1663.64 a	6582.30±748.78 b
C_3_N_4_	21.55±5.62 ab	23.29±3.59 b	5985.90±1560.16 ab	6468.70±996.87 b
Fe_2_O_3_/g-C_3_N_4_	22.50±6.12 a	28.00±1.63 a	6249.80±1699.73 a	7777.60±453.60 a
Irrigation level				
100%ET	28.74±1.56 a	27.47±1.65 a	7984.70±434.02 a	7629.72±459.89 a
75%ET	21.21±2.02 b	23.94±2.81 b	5891.00±562.61 b	6649.35±780.11 b
50%ET	15.04±1.70 c	20.91±3.23 c	4178.10±472.54 c	5806.94±896.72 c
Spraying treatment×Irrigation level				
WT×100%ET	26.67±0.85	25.69±0.31 cd	7407.20±236.03	7136.20±86.60 cd
WT×75%ET	20.17±1.43	20.89±0.31 f	5601.70±398.24	5802.30±87.29 f
WT×50%ET	13.67±1.43	17.71±0.32 g	3796.20±398.24	4919.60±88.59 g
FeSO4×100%ET	30.33±0.87	26.69±0.02 bc	8425.70±242.51	7414.00±7.14 bc
FeSO4×75%ET	20.83±1.25	24.22±0.23 de	5786.90±346.44	6728.20±62.95 de
FeSO4×50%ET	16.17±1.03	20.18±0.35 f	4490.60±285.38	5604.80±97.21 f
C_3_N_4_×100%ET	28.65±0.83	27.49±0.63 b	7958.10±231.57	7635.60±175.24 b
C_3_N_4_×75%ET	20.83±1.43	22.86±2.45 e	5786.90±398.24	6351.10±681.94 e
C_3_N_4_×50%ET	15.17±0.47	19.51±0.35 f	4212.80±130.94	5419.60±97.21 f
Fe_2_O_3_/g-C_3_N_4_×100%ET	29.33±0.62	30.00±0.54 a	8147.90±173.22	8333.10±151.20 a
Fe_2_O_3_/g-C_3_N_4_×75%ET	23.00±2.48	27.78±0.31 b	6388.70±689.78	7715.80±87.29 b
Fe_2_O_3_/g-C_3_N_4_×50%ET	15.17±2.25	26.22±0.63 bc	4212.80±624.55	7283.70±174.59 bc
Significance				
Spraying treatment	*	**	*	**
Irrigation level	**	**	**	**
Spraying treatment×Irrigation level	ns	**	ns	**

ns, *, ** non-significant or significant at p ≤ 0.05 and 0.01, respectively. Values represent means ± standard deviation of three independent replications (n = 3). Different letters within the same column indicate significant differences at p ≤ 0.05 among the treatments, according to Duncan’s multiple range test. The abbreviation of features name: C_3_N_4_, carbon nitride; Fe_2_O_3_/g-C_3_N_4_, Fe_2_O_3_-graphitic carbon nitride nanostructure; 100%ET, normal condition; 75%ET, moderate drought stress; 50%ET, severe drought stress; WT, water (Control).

### 3.4 Water use efficiency highly increases under Fe_2_O_3_/g-C_3_N_4_ treatment

The interaction effect of spraying and irrigation level was significant in 2023 for both WUE characteristics, as can be seen in **Table 5**. In 50%ET-irrigated plants, WUE in fruits and oil enhanced by 2% and 25% in 2022 and 38 and 80% in 2023, respectively. Furthermore, the olive plants treated with Fe_2_O_3_/g-C_3_N_4_ displayed an increase in WUE in fruit and in oil by 10 and 120% in 2022 and 15 and 55% in 2023, respectively, compared to non-treated plants. It is valuable to point out that while other treatments were effective, the Fe_2_O_3_/g-C_3_N_4_ one outperformed the others under severe drought.

**Table 5:** The effect of spraying treatment, irrigation level and their interactions on water use efficiency in fruit and water use efficiency in oil in *Olea europaea* var. Shengeh.

Source of variance	Water use efficiency in fruit (Kg.m^-3^)		Water use efficiency in oil (Kg.m^-3^)	
	2022	2023	2022	2023
Spraying treatment				
WT	0.77±0.06	0.73±0.10 c	0.05±0.01 c	0.07±0.02 c
FeSO_4_	0.87±0.07	0.81±0.14 b	0.07±0.01 b	0.08±0.03 b
C_3_N_4_	0.83±0.05	0.79±0.13 b	0.07±0.01 b	0.06±0.02 c
Fe_2_O_3_/g-C_3_N_4_	0.87±0.09	0.88±0.11 a	0.09±0.02 a	0.09±0.04 a
Irrigation level				
100%ET	0.83±0.04	0.67±0.04 c	0.05±0.01 c	0.05±0.01 c
75%ET	0.81±0.08	0.78±0.09 b	0.07±0.02 b	0.06±0.01 b
50%ET	0.86±0.10	0.95±0.05 a	0.08±0.02 a	0.11±0.02 a
Spraying treatment×Irrigation level				
WT×100%ET	0.77±0.02	0.63±0.01 g	0.04±0.01	0.05±0.01 hi
WT×75%ET	0.77±0.05	0.69±0.01 f	0.05±0.01	0.06±0.01 fg
WT×50%ET	0.79±0.08	0.87±0.01 c	0.05±0.01	0.09±0.01 cd
FeSO4×100%ET	0.87±0.02	0.65±0.01 fg	0.06±0.01	0.06±0.01 fgh
FeSO4×75%ET	0.80±0.05	0.79±0.01 d	0.07±0.01	0.07±0.01 ef
FeSO4×50%ET	0.93±0.06	0.99±0.02 a	0.09±0.01	0.12±0.01 b
C_3_N_4_×100%ET	0.83±0.02	0.67±0.01 fg	0.05±0.01	0.04±0.01 i
C_3_N_4_×75%ET	0.80±0.05	0.75±0.08 de	0.07±0.01	0.05±0.01 gh
C_3_N_4_×50%ET	0.87±0.03	0.96±0.02 ab	0.09±0.01	0.10±0.01 c
Fe_2_O_3_/g-C_3_N_4_×100%ET	0.84±0.02	0.74±0.01 e	0.07±0.01	0.06±0.01 fgh
Fe_2_O_3_/g-C_3_N_4_×75%ET	0.89±0.09	0.91±0.01 bc	0.09±0.01	0.08±0.01 de
Fe_2_O_3_/g-C_3_N_4_×50%ET	0.87±0.13	1.00±0.00 a	0.11±0.01	0.14±0.01 a
Significance				
Spraying treatment	ns	**	**	**
Irrigation level	ns	**	**	**
Spraying treatment×Irrigation level	ns	**	ns	**

ns, *, ** non-significant or significant at p ≤ 0.05 and 0.01, respectively. Values represent means ± standard deviation of three independent replications (n = 3). Different letters within the same column indicate significant differences at p ≤ 0.05 among the treatments, according to Duncan’s multiple range test. The abbreviation of features name: C_3_N_4_, carbon nitride; Fe_2_O_3_/g-C_3_N_4_, Fe_2_O_3_-graphitic carbon nitride nanostructure; 100%ET, normal condition; 75%ET, moderate drought stress; 50%ET, severe drought stress; WT, water (Control).

### 3.5 Fe_2_O_3_/g-C_3_N_4_ treatment reshapes antioxidant response and reduces drought damage

As seen in **Table 6**, under severe drought stress, POD and CAT showed activities considerably higher than those in normal conditions, with increases of 182% and 254% in 2022, and 229% and 470% in 2023, respectively. Under 50%ET, the use of Fe_2_O_3_/g-C_3_N_4_ further increased the activity of antioxidant enzymes, particularly POD and CAT by 59% and 61% in 2022, and 68% and 146% in 2023, respectively, compared to the controls. On the contrary, RWC in olive plants subjected to severe drought stress decreased in a severity-dependent manner by 26% in 2022 and by 29% in 2023 over 100%ET; while the use of Fe_2_O_3_/g-C_3_N_4_ ameliorated this decrease, determining an improvement of RWC by 41% and 4% in 2022 and 2023, respectively, compared to the non-treated stress control. Similar to response of enzymatic antioxidants, EL increased due to severe drought stress by 70% and 71% in the consecutive years, respectively, compared to the normal conditions. However, the EL of the olive plants treated by Fe_2_O_3_/g-C_3_N_4_ decreased by 26% in 2022 and 18% in 2023, compared to that of the non-treated plants (**Table 6**).

**Table 6:** The effect of spraying treatment, irrigation level and their interactions on POD, CAT, RWC and EL in leaves of *Olea europaea* var. Shengeh.

Source of variance	POD (units.mg^-1^protein)		CAT (units.mg^-1^protein)		RWC (%)		EL (%)	
	2022	2023	2022	2023	2022	2023	2022	2023
Spraying treatment								
WT	1.78±0.80 c	1.84±0.88 c	0.72±0.38 c	0.52±0.34 c	55.94±7.55 c	60.36±9.17 bc	34.67±7.80 a	30.09±6.77 b
FeSO_4_	2.21±1.07 b	2.38±1.09 b	0.94±0.54 b	0.85±0.37 b	63.56±7.41 b	65.11±9.21 a	28.78±7.95 b	29.43±6.44 b
C_3_N_4_	2.01±0.98 bc	2.22±0.95 bc	0.85±0.49 bc	0.79±0.36 b	62.21±9.19 b	62.49±9.27 ab	28.05±6.31 b	27.64±6.38 b
Fe_2_O_3_/g-C_3_N_4_	2.85±1.21 a	3.21±1.38 a	1.53±0.49 a	1.82±0.55 a	73.78±7.08 a	58.00±8.60 c	22.96±7.38 c	37.72±7.62 a
Irrigation level								
100%ET	1.24±0.30 c	1.22±0.31 c	0.54±0.25 c	0.57±0.44 c	72.79±5.62 a	72.95±3.46 a	20.03±4.17 c	23.17±4.69 c
75%ET	1.90±0.58 b	2.37±0.80 b	0.89±0.44 b	0.92±0.56 b	62.75±7.35 b	59.51±3.75 b	28.89±5.39 b	32.07±4.79 b
50%ET	3.50±0.70 a	3.66±0.78 a	1.61±0.36 a	1.50±0.54 a	56.07±8.97 c	52.01±4.01 c	36.92±5.12 a	38.43±4.65 a
Spraying treatment×Irrigation level								
WT×100%ET	0.98±0.13	0.87±0.16	0.35±0.04	0.17±0.03	65.50±1.08	72.13±0.63	25.33±1.25	22.03±0.05
WT×75%ET	1.59±0.43	1.80±0.42	0.57±0.03	0.41±0.03	54.17±1.18	57.93±2.19	35.67±3.09	30.57±0.42
WT×50%ET	2.77±0.30	2.86±0.40	1.24±0.03	0.97±0.05	48.17±3.66	51.00±3.89	43.00±3.74	37.67±3.86
FeSO4×100%ET	1.23±0.13	1.19±0.07	0.44±0.01	0.49±0.06	73.00±1.41	77.00±0.00	20.48±1.44	21.73±1.52
FeSO4×75%ET	1.82±0.47	2.27±0.56	0.70±0.07	0.73±0.04	61.50±1.08	63.00±2.16	27.57±4.74	29.77±1.25
FeSO4×50%ET	3.60±0.37	3.67±0.38	1.68±0.17	1.35±0.09	56.17±3.66	55.33±2.87	38.28±2.09	36.80±2.66
C_3_N_4_×100%ET	1.09±0.09	1.14±0.05	0.40±0.02	0.42±0.05	73.00±1.63	74.33±0.47	20.21±1.29	19.57±1.38
C_3_N_4_×75%ET	1.68±0.44	2.23±0.54	0.64±0.04	0.68±0.01	61.67±0.47	60.43±2.54	29.28±1.26	28.60±0.43
C_3_N_4_×50%ET	3.26±0.39	3.30±0.29	1.52±0.16	1.28±0.07	51.97±5.36	52.70±3.29	34.67±3.09	34.77±1.76
Fe_2_O_3_/g-C_3_N_4_×100%ET	1.67±0.23	1.67±0.12	0.96±0.04	1.20±0.43	79.67±4.50	68.33±2.62	14.11±0.95	29.33±5.44
Fe_2_O_3_/g-C_3_N_4_×75%ET	2.50±0.51	3.17±0.90	1.63±0.12	1.87±0.09	73.67±4.19	56.67±4.11	23.04±0.86	39.33±4.19
Fe_2_O_3_/g-C_3_N_4_×50%ET	4.40±0.43	4.80±0.00	2.00±0.41	2.39±0.07	68.00±6.68	49.00±2.94	31.74±2.52	44.50±2.86
Significance								
Spraying treatment	**	**	**	**	**	**	**	**
Irrigation level	**	**	**	**	**	**	**	**
Spraying treatment×Irrigation level	ns	ns	ns	ns	ns	ns	ns	ns

ns, *, ** non-significant or significant at p ≤ 0.05 and 0.01, respectively. Values represent means ± standard deviation of three independent replications (n = 3). Different letters within the same column indicate significant differences at p ≤ 0.05 among the treatments, according to Duncan’s multiple range test. The abbreviation of features name: C_3_N_4_, carbon nitride; Fe_2_O_3_/g-C_3_N_4_, Fe_2_O_3_-graphitic carbon nitride nanostructure; 100%ET, normal condition; 75%ET, moderate drought stress; 50%ET, severe drought stress; WT, water (Control); POD, peroxidase activity; CAT, catalase activity; RWC, relative water content; EL, electrolyte leakage.

### 3.6 Shifts in nutritional elements under stress and treatments

As shown in **Table 7**, Ca^2+^ and K^+^ in the olive plants exposed to severe drought stress declined by 50% and 83% in 2022, and by 46 and 24% in 2023, respectively, compared to 100%ET. Whereas, Na^+^ increased in plants under severe drought stress by 48% and 57% in the successive experimental years, respectively, as compared to normal conditions. The application of Fe_2_O_3_/g-C_3_N_4_ significantly enhanced the contents of Ca^2+^ and K^+^ by 89% and 600% in 2022, and 114 and 138% in 2023, respectively. This beneficial treatment also led to a significant decline of Na^+^ by 30% and 2% in 2022 and 2023, respectively, compared to controls.

**Table 7:** The effect of spraying treatment, irrigation level and their interactions on Ca^2+^, Na^+^ and K^+^ in leaves of *Olea europaea* var. Shengeh.

Source of variance	Ca^2+^ (%)		Na^+^ (%)		K^+^ (%)	
	2022	2023	2022	2023	2022	2023
Spraying treatment						
WT	1.57±0.48 b	1.61±0.39 b	0.34±0.07 a	0.34±0.08 a	0.88±0.52 c	0.91±0.17 c
FeSO_4_	1.91±0.45 b	1.90±0.47 b	0.24±0.07 bc	0.29±0.08 b	1.28±0.57 b	1.25±0.16 b
C_3_N_4_	1.77±0.46 b	1.83±0.48 b	0.26±0.18 b	0.26±0.07 bc	1.00±0.47 c	1.13±0.18 b
Fe_2_O_3_/g-C_3_N_4_	2.78±0.87 a	2.74±0.79 a	0.19±0.08 c	0.23±0.17 c	2.14±0.44 a	2.34±0.47 a
Irrigation level						
100%ET	2.57±0.57 a	2.44±0.37 a	0.16±0.09 c	0.19±0.08 c	1.83±0.55 a	1.63±0.74 a
75%ET	2.02±0.68 b	2.04±0.60 a	0.23±0.09 b	0.26±0.07 b	1.35±0.53 b	1.38±0.55 b
50%ET	1.44±0.51 c	1.59±0.78 b	0.38±0.09 a	0.39±0.10 a	0.80±0.61 c	1.21±0.45 c
Spraying treatment×Irrigation level						
WT×100%ET	2.13±0.20	2.07±0.12	0.29±0.02	0.28±0.02 bc	1.44±0.29	1.04±0.13 de
WT×75%ET	1.53±0.18	1.65±0.05	0.32±0.03	0.32±0.04 b	0.94±0.09	0.91±0.19 e
WT×50%ET	1.06±0.18	1.12±0.02	0.43±0.04	0.44±0.04 a	0.25±0.11	0.79±0.04 e
FeSO4×100%ET	2.39±0.15	2.30±0.22	0.19±0.04	0.24±0.02 bc	1.84±0.46	1.37±0.15 d
FeSO4×75%ET	1.89±0.29	1.93±0.32	0.20±0.02	0.27±0.06 bc	1.30±0.25	1.30±0.03 d
FeSO4×50%ET	1.45±0.27	1.48±0.43	0.34±0.04	0.35±0.11 ab	0.70±0.25	1.07±0.06 de
C_3_N_4_×100%ET	2.26±0.27	2.40±0.12	0.08±0.04	0.20±0.01 c	1.54±0.22	1.24±0.11 d
C_3_N_4_×75%ET	1.81±0.09	1.77±0.31	0.25±0.13	0.25±0.05 bc	1.00±0.11	1.06±0.13 de
C_3_N_4_×50%ET	1.25±0.17	1.34±0.09	0.46±0.10	0.34±0.05 ab	0.48±0.14	1.09±0.22 de
Fe_2_O_3_/g-C_3_N_4_×100%ET	3.50±0.00	3.00±0.00	0.10±0.03	0.07±0.02 d	2.50±0.41	2.87±0.19 a
Fe_2_O_3_/g-C_3_N_4_×75%ET	2.85±0.84	2.82±0.62	0.17±0.02	0.19±0.05 c	2.17±0.31	2.27±0.20 b
Fe_2_O_3_/g-C_3_N_4_×50%ET	2.00±0.64	2.40±1.14	0.30±0.02	0.43±0.13 a	1.77±0.20	1.88±0.29 c
Significance						
Spraying treatment	**	**	**	**	**	**
Irrigation level	**	**	**	**	**	**
Spraying treatment×Irrigation level	ns	ns	ns	*	ns	**

ns, *, ** non-significant or significant at p ≤ 0.05 and 0.01, respectively. Values represent means ± standard deviation of three independent replications (n = 3). Different letters within the same column indicate significant differences at p ≤ 0.05 among the treatments, according to Duncan’s multiple range test. The abbreviation of features name: C_3_N_4_, carbon nitride; Fe_2_O_3_/g-C_3_N_4_, Fe_2_O_3_-graphitic carbon nitride nanostructure; 100%ET, normal condition; 75%ET, moderate drought stress; 50%ET, severe drought stress; WT, water (Control); Ca^2+^, calcium; Na^+^, sodium; K^+^, potassium.

### 3.7 Fe_2_O_3_/g-C_3_N_4_ treatment boosts photosynthetic pigments

Chl a, Chl b and Chl a+b decreased as affected by drought stress depending on severity level by 74%, 27% and 79% in 2022 and 79, 47 and 83% in 2023, irrespective of treatment as compared with normal conditions. Furthermore, the treatment with Fe_2_O_3_/g-C_3_N_4_ increased the contents of Chl a, Chl b and Chl a+b by about 8-fold, 2-fold and 2-fold, respectively, in 2022 respectively. In 2023, this observed enhancement of Chl a, Chl b and Chl a+b was up to 8-fold, up to 4-fold and nearly 5-fold respectively. In 2022 and 2023, the use of Fe_2_O_3_/g-C_3_N_4_ caused to decline in Chl a/Chl b by 22 and 51% respectively in normal conditions while in severe drought stress, the use of Fe_2_O_3_/g-C_3_N_4_ led to enhance of Chl a/Chl b by 177 and 45% in 2022 and 2023 respectively (**Table 8**).

**Table 8:** The effect of spraying treatment, irrigation level and their interactions on Chl a, Chl b, Chl a+b and Chl a/Chl b in leaves *Olea europaea* var. Shengeh.

Source of variance	Chl a (mg.g^-1^FW)		Chl b (mg.g^-1^FW)		Chl a+b (mg.g^-1^FW)		Chl a/Chl b	
	2022	2023	2022	2023	2022	2023	2022	2023
Spraying treatment								
WT	0.37±0.20 d	0.41±0.23 c	0.12±0.05 d	0.10±0.05 d	0.84±0.46 c	0.82±0.48 c	3.30±1.92	4.33±2.35 a
FeSO_4_	1.13±0.33 b	0.98±0.36 b	0.32±0.12 b	0.41±0.14 b	1.05±0.49 b	1.05±0.51 b	3.71±1.06	2.43±0.63 b
C_3_N_4_	0.89±0.34 c	0.83±0.38 b	0.23±0.10 c	0.30±0.08 c	0.96±0.49 bc	0.99±0.52 bc	4.35±1.88	2.79±1.23 b
Fe_2_O_3_/g-C_3_N_4_	1.67±0.22 a	1.61±0.23 a	0.44±0.09 a	0.57±0.13 a	1.60±0.61 a	1.98±0.60 a	3.95±0.94	2.95±0.74 b
Irrigation level								
100%ET	1.26±0.46 a	1.22±0.41 a	0.35±0.16 a	0.44±0.21 a	1.71±0.38 a	1.82±0.48 a	3.93±1.12	3.30±1.46
75%ET	1.06±0.50 b	1.02±0.48 b	0.29±0.14 b	0.35±0.20 b	1.12±0.32 b	1.19±0.48 b	3.96±1.62	3.60±1.92
50%ET	0.72±0.53 c	0.64±0.52 c	0.19±0.10 c	0.25±0.13 c	0.51±0.28 c	0.62±0.52 c	3.59±1.82	2.47±1.01
Spraying treatment×Irrigation level								
WT×100%ET	0.62±0.07	0.71±0.07	0.15±0.04	0.15±0.04	1.40±0.01	1.40±0.07	4.63±1.47	5.23±1.71
WT×75%ET	0.33±0.02	0.37±0.01	0.11±0.04	0.09±0.04	0.84±0.07	0.81±0.06	3.70±2.10	5.37±2.94
WT×50%ET	0.16±0.04	0.15±0.05	0.11±0.04	0.08±0.05	0.29±0.09	0.24±0.08	1.58±0.33	2.41±0.73
FeSO4×100%ET	1.40±0.14	1.25±0.23	0.44±0.07	0.53±0.12	1.57±0.05	1.66±0.10	3.29±0.57	2.37±0.23
FeSO4×75%ET	1.22±0.13	1.09±0.25	0.35±0.06	0.42±0.10	1.17±0.10	1.07±0.13	3.52±0.48	2.62±0.42
FeSO4×50%ET	0.79±0.32	0.60±0.20	0.18±0.02	0.28±0.04	0.41±0.09	0.44±0.12	4.32±1.59	2.29±1.02
C_3_N_4_×100%ET	1.16±0.17	1.17±0.20	0.30±0.09	0.39±0.01	1.52±0.09	1.60±0.10	4.18±1.25	3.04±0.65
C_3_N_4_×75%ET	1.02±0.19	0.99±0.18	0.25±0.09	0.29±0.05	0.98±0.16	1.00±0.15	4.79±2.38	3.64±1.41
C_3_N_4_×50%ET	0.48±0.17	0.35±0.05	0.14±0.04	0.22±0.05	0.37±0.15	0.37±0.10	4.08±2.10	1.70±0.59
Fe_2_O_3_/g-C_3_N_4_×100%ET	1.87±0.05	1.75±0.18	0.53±0.07	0.69±0.08	2.35±0.12	2.62±0.10	3.62±0.65	2.57±0.46
Fe_2_O_3_/g-C_3_N_4_×75%ET	1.67±0.12	1.62±0.22	0.44±0.05	0.59±0.07	1.49±0.36	1.90±0.43	3.84±0.78	2.77±0.60
Fe_2_O_3_/g-C_3_N_4_×50%ET	1.47±0.20	1.47±0.19	0.35±0.04	0.43±0.05	0.97±0.05	1.43±0.42	4.38±1.28	3.50±0.85
Significance								
Spraying treatment	**	**	**	**	**	**	ns	*
Irrigation level	**	**	**	**	**	**	ns	ns
Spraying treatment×Irrigation level	ns	ns	ns	ns	ns	ns	ns	ns

ns, *, ** non-significant or significant at p ≤ 0.05 and 0.01, respectively. Values represent means ± standard deviation of three independent replications (n = 3). Different letters within the same column indicate significant differences at p ≤ 0.05 among the treatments, according to Duncan’s multiple range test. The abbreviation of features name: C_3_N_4_, carbon nitride; Fe_2_O_3_/g-C_3_N_4_, Fe_2_O_3_-graphitic carbon nitride nanostructure; 100%ET, normal condition; 75%ET, moderate drought stress; 50%ET, severe drought stress; WT, water (Control); Chl a, chlorophyll a; Chl b, chlorophyll b.

### 3.8 Both stress and Fe_2_O_3_/g-C_3_N_4_ treatment modulate osmolytes and antioxidant profiles

As observed in **Table 9**, in the olive plants exposed to severe drought stress without treatment, proline remarkably decreased by 74% and 81% in the two consecutive years, respectively, as compared with 100%ET, whereas NSC content, total phenolic content and MDA enhanced by 61%, 86% and 111% in 2022, and 78%, 171% and 52% in 2023, respectively. In the olive plants treated by Fe_2_O_3_/g-C_3_N_4_, proline, NSC content and MDA decreased by 67%, 42% and 18% in 2022, and 64%, 39% and 64% in 2023, respectively, while total phenolic content of the plant under severe drought stress increased by 85% and 162% in two successive years, respectively, when treated with Fe_2_O_3_/g-C_3_N_4_ compared to no treatment (**Table 9**).

**Table 9:** The effect of spraying treatment, irrigation level and their interactions on proline content, soluble carbohydrate content or none structural carbohydrates (NSC), total phenolic content and MDA in leaves of *Olea europaea* var. Shengeh.

Source of variance	Proline content (µg.g^-1^FW)		NSC (mg.g^-1^FW)		Total phenolic content (mg.100g^-1^FW)		MDA (nmol.g^-1^FW)	
	2022	2023	2022	2023	2022	2023	2022	2023
Spraying treatment								
WT	38.33±18.49 a	35.56±20.72 a	13.67±4.47 a	17.61±4.46 a	52.11±15.57 d	34.44±15.00 d	1.82±0.62 a	3.31±0.71 a
FeSO_4_	30.56±18.80 b	30.78±21.23 ab	8.89±3.98 c	10.33±3.31 b	87.44±19.19 b	97.11±24.68 b	1.13±0.68 bc	1.08±0.36 c
C_3_N_4_	34.17±18.24 ab	35.56±21.19 a	11.22±3.52 b	11.55±3.53 b	71.89±19.32 c	78.11±19.66 c	1.33±0.57 b	2.38±0.76 b
Fe_2_O_3_/g-C_3_N_4_	20.22±15.13 c	27.11±18.78 b	7.67±4.11 d	8.33±3.77 c	111.11±21.83 a	128.56±19.52 a	0.91±0.83 c	0.94±0.69 c
Irrigation level								
100%ET	53.00±8.86 a	57.00±7.20 a	7.83±3.89 c	8.21±3.24 c	63.58±21.96 c	62.92±34.82 c	0.68±0.42 c	1.45±0.89 b
75%ET	27.87±10.15 b	31.00±8.42 b	10.50±4.29 b	11.17±4.55 b	74.08±23.17 b	88.58±39.00 b	1.06±0.49 b	1.79±1.11 b
50%ET	11.58±5.04 c	8.75±3.16 c	12.75±4.36 a	16.50±3.55 a	104.25±24.16 a	102.17±34.03 a	2.15±0.38 a	2.54±1.21 a
Spraying treatment×Irrigation level								
WT×100%ET	58.33±6.24	62.00±4.32	10.33±3.30	12.33±2.05	39.67±1.25	19.67±0.94	1.17±0.23	2.63±0.38
WT×75%ET	41.33±5.91	32.67±0.94	14.00±3.74	18.50±1.22	42.67±1.25	30.33±8.99	1.83±0.23	3.30±0.50
WT×50%ET	15.33±3.68	12.00±2.45	16.67±3.86	22.00±2.45	74.00±0.82	53.33±1.25	2.47±0.45	4.00±0.41
FeSO4×100%ET	55.00±6.38	58.00±1.41	7.00±3.56	7.17±1.54	69.00±1.41	68.33±2.36	0.60±0.08	0.80±0.08
FeSO4×75%ET	25.33±2.87	25.33±10.14	8.67±3.77	9.17±0.23	82.00±6.68	101.00±7.79	0.77±0.05	0.92±0.03
FeSO4×50%ET	11.33±4.19	9.00±1.41	11.00±3.56	14.67±0.47	111.33±10.87	122.00±17.28	2.03±0.38	1.53±0.29
C_3_N_4_×100%ET	58.00±4.55	58.00±9.90	8.33±3.30	8.33±2.05	52.33±12.55	53.67±13.20	0.89±0.07	1.83±0.40
C_3_N_4_×75%ET	29.83±2.72	39.00±7.87	11.67±2.49	10.33±1.25	68.33±2.62	87.33±5.25	1.02±0.13	2.13±0.61
C_3_N_4_×50%ET	14.67±1.70	9.67±0.94	13.67±2.36	16.00±0.00	95.00±5.10	93.33±6.60	2.09±0.28	3.17±0.45
Fe_2_O_3_/g-C_3_N_4_×100%ET	40.67±2.05	50.00±3.56	5.67±3.77	5.00±1.63	93.33±12.47	110.00±21.60	0.08±0.05	0.53±0.19
Fe_2_O_3_/g-C_3_N_4_×75%ET	15.00±2.16	27.00±1.63	7.67±3.77	6.67±0.94	103.33±12.47	135.67±12.28	0.63±0.12	0.83±0.54
Fe_2_O_3_/g-C_3_N_4_×50%ET	5.00±0.82	4.33±0.47	9.67±3.77	13.33±0.47	136.67±9.43	140.00±0.00	2.03±0.17	1.45±0.82
Significance								
Spraying treatment	**	*	**	**	**	**	**	**
Irrigation level	**	**	**	**	**	**	**	**
Spraying treatment×Irrigation level	ns	ns	ns	ns	ns	ns	ns	ns

ns, *, ** non-significant or significant at p ≤ 0.05 and 0.01, respectively. Values represent means ± standard deviation of three independent replications (n = 3). Different letters within the same column indicate significant differences at p ≤ 0.05 among the treatments, according to Duncan’s multiple range test. The abbreviation of features name: C_3_N_4_, carbon nitride; Fe_2_O_3_/g-C_3_N_4_, Fe_2_O_3_-graphitic carbon nitride nanostructure; 100%ET, normal condition; 75%ET, moderate drought stress; 50%ET, severe drought stress; WT, water (Control); NSC, soluble carbohydrate content or none structural carbohydrates; MDA, malondialdehyde.

### 3.9 Statistical analyses reveal interactions between treatments and water regimes

**Figure 5:**
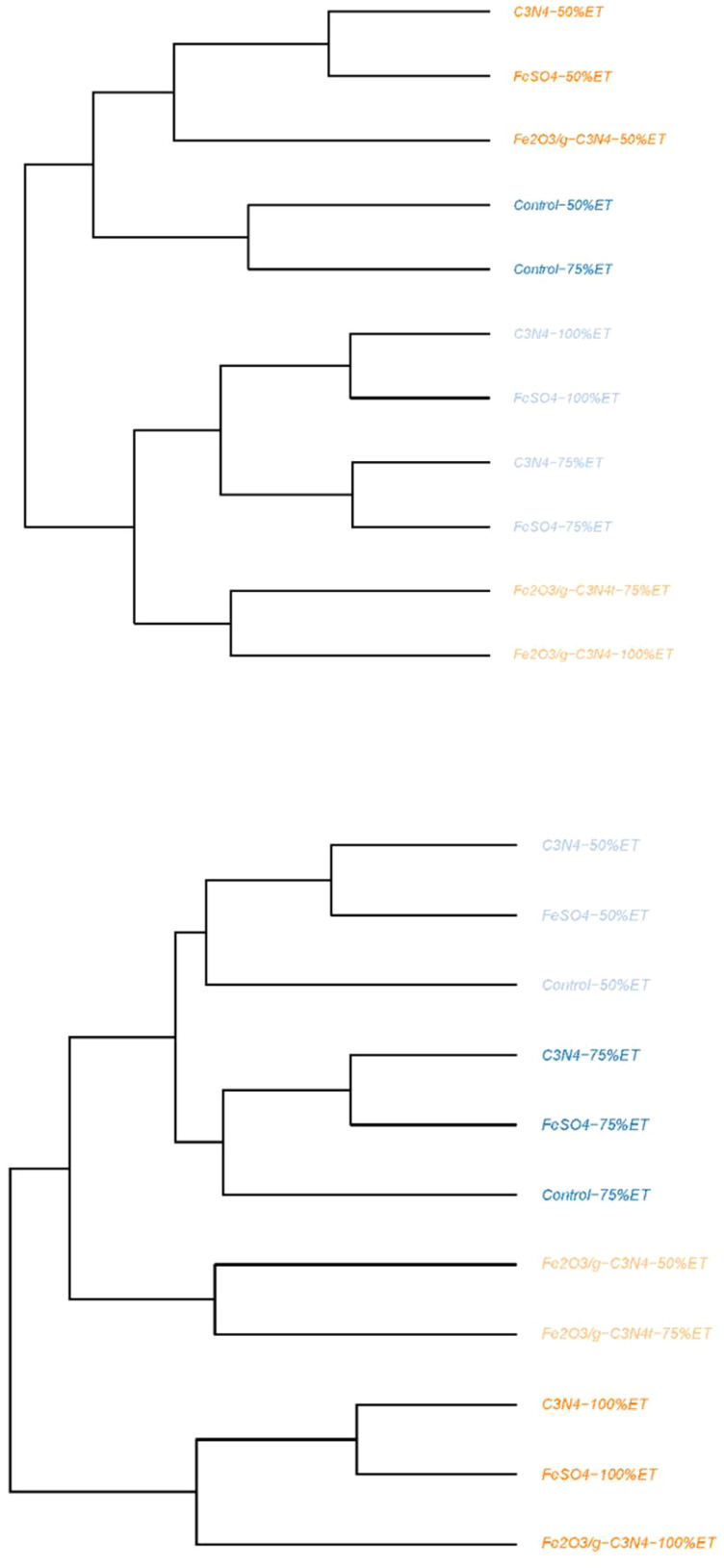
Dendrogram clustering of treated olive trees growing in normal conditions (100%ET), moderate drought stress (75%ET) and severe drought stress (50%ET) and dendrogram clustering and correlation of all assayed pomological, physiological and yield characteristics in Control, FeSO_4_, C_3_N_4_ and Fe_2_O_3_/g-C_3_N_4_ treated plants in “Shengeh” olive cultivar (Top: The year 2022; Bottom: The year 2023).

In the first experimental year, dendrogram clustering separated control and treated olive trees growing under three water regimes in two main clusters. The first main cluster consisted of two sub-clusters. The first sub-cluster included C_3_N_4_ and FeSO_4_ with 50%ET and Fe_2_O_3_/g-C_3_N_4_-50%ET. The second sub-cluster contained control with 50% and 75%ET. The second major group also consisted of two sub-clusters. The first one included C_3_N_4_ as well as FeSO_4_ with 100%ET and C_3_N_4_ and FeSO_4_ with 75%ET. The second sub-cluster included Fe_2_O_3_/g-C_3_N_4_ with 75 and 100%ET. In the second experimental year, dendrogram clustering separated control and foliar-sprayed olive trees under 3 water regimes into two main groups. The first major cluster included two sub-clusters. The first one consisted of C_3_N_4_, FeSO_4_ and control with 50% and 75%ET. The second one included Fe_2_O_3_/g-C_3_N_4_ with 50% and 75%ET. The second main group contained C_3_N_4_, FeSO_4_ and Fe_2_O_3_/g-C_3_N_4_ with 100%ET (**Figure 5**).

**Figure 6:**
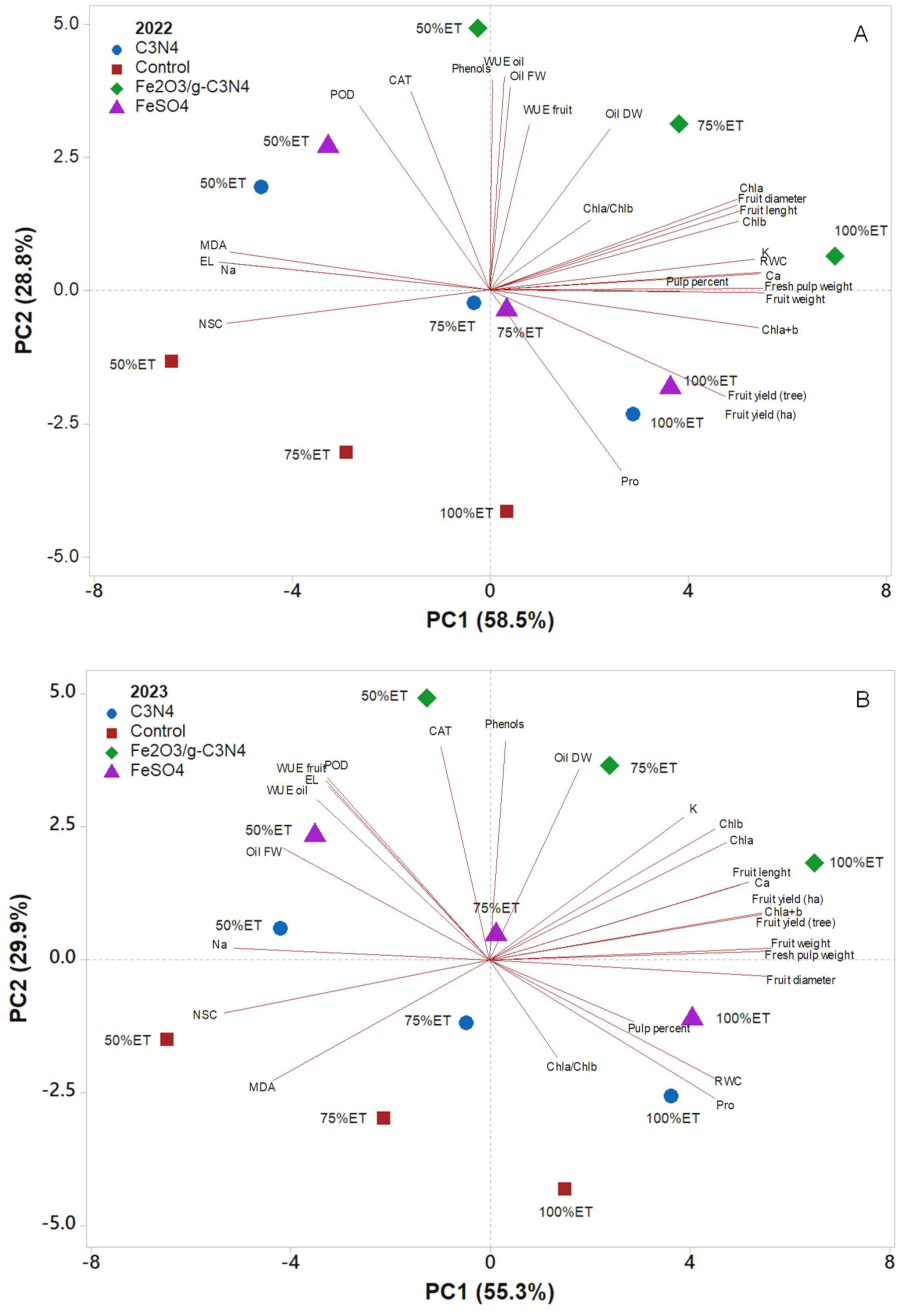
Principal component analysis (PCA) of pomological, yield, and physiological parameters of “Shengeh” olive cultivar grown under different drought stress conditions (100%, 75%, and 50% ET) over the two experimental years 2022 **(A)** and 2023 **(B)**. The analysis compared the effects of exogenous foliar application of Fe_2_O_3_-modified carbon nitride nanostructures with control, FeSO_4_, and C_3_N_4_ treatments.

Foliar application treatments and different watering levels were separated by applying a principal component analysis (PCA) for the two experimental years (2022 and 2023) (**Figure 6**). The first two principal components (PCs) were related with eigenvalues higher than 1, explaining a total variance of 87.4% in the first experimental year (PC1 = 58.5% and PC2 = 28.8%), and 85.2% in the second year (PC1 = 55.3% and PC2 = 29.9%). In both years, the irrigation regimes contributed to the clear separation on PC1, while the foliar spray treatments contributed to the separation on PC2. PC1 was positively correlated to fruit diameter, fruit length, fruit weight, fresh pulp weight, RWC, Chla+b, Ca, K, Chla, Chlb, fruit yield (ha), and fruit yield (tree), which clustered with Fe_2_O_3_/g-C_3_N_4_ treatments under 100%ET and 75%ET in the upper right quadrant, and FeSO_4_ under 100%ET in the lower right quadrant for both years (**Figures 6A, B**). Whereas PC1 was negatively correlated to Na and NSC in both years, and also to EL and MDA specifically in 2022, and clustering C_3_N_4_ under 50%ET and control under 50%ET in the upper and lower left quadrants, respectively (**Figures 6A, B**). PC2 was positively correlated to WUE oil, phenols, oil FW, CAT, POD, WUE, fruit and oil DW, clustering with Fe_2_O_3_/g-C_3_N_4_ treatment under 50%ET, in 2022. Similarly, in 2023, PC2 was positively correlated with phenols, CAT, oil DW and POD, clustering with Fe_2_O3/g-C_3_N_4_ under 50%ET. On the contrary, PC2 was negatively correlated with proline, clustering with C_3_N_4_ in the lower right quadrant in both years. Additionally, in 2023, PC2 was negatively correlated also with MDA, clustering with control treatment under 75%ET in the lower left quadrant.

## 4 Discussion

In Mediterranean-type regions, identified as climate hotspots, drought conditions are exacerbated by hot and dry weather during the spring and summer, high rates of evapotranspiration, and low rainfall, including during the winter months (Gholami et al., 2022). Olive trees in these semi-arid environments have developed various mechanisms to cope with drought. These mechanisms involve a range of strategies and adaptive changes that require consuming significant amounts of energy and carbon skeletons, ultimately hindering vegetative growth, physiological functions, and reproduction, which leads to reduced crop yields (Fahad et al., 2017). In this study, we have investigated the beneficial effects of applying exogenous foliar Fe_2_O_3_ modified carbon nitride nanostructures, in comparison with control, FeSO_4_, and C_3_N_4_ treatments, on “Shengeh” olive cultivars grown under different water deficit conditions (100%, 75%, and 50% ET) over two experimental years (2022 and 2023).

Indeed, in this experimental work, we found a decrease in pomological characteristics as a consequence of the water deficit. In fact, drought, leads to the closure of stomata to reduce water loss, which in turn indirectly decreases the intake of carbon dioxide into leaves. This plant strategy affects photosynthesis, resulting in reduced vegetative growth (Lahive et al., 2018). In particular, the reduced carbon dioxide in the mesophyll tissue slows down the Calvin cycle (Wang et al., 2018) and subsequently limits the use of light-generated photosynthetic products, such as ATP and NADPH. This causes a loss of efficiency in the photosynthetic electron transport chain, leading to photooxidative stress, which destroys photosynthetic pigments and causes the thylakoid structure to deteriorate. Consequently, there is a further decline in overall photosynthetic efficiency and growth capacity. Plants try to cope with water deficiency mainly by reducing the water loss through transpiration, thereby and with this change, increasing the plant’s WUE (Kapoor et al., 2020), as observed in this study. In light of the previous considerations, in well-irrigated plants, growth improvement occurs for two reasons. First, increased turgor pressure and RWC due to sufficient water uptake result in cell enlargement. Secondly, stomata opening leads to improved CO_2_ absorption and assimilation, actively enhancing photosynthesis. Consequently, and eventually, more assimilates contribute to cell growth (Nxele et al., 2017). Indeed, under drought stress, protective metabolites and/or osmolytes are synthesized and accumulated to provide antioxidant response and osmoregulation. Phenolic compounds are vital antioxidants that protect plant cells from oxidative damage. Particularly, they protect membranes from lipid peroxidation by inhibiting the initiation and propagation of oxidation chain reactions (Gao et al., 2018). Their action is attributable to blocking oxidative reactions, reducing hydrogen levels, scavenging free radicals, increasing peroxidase enzyme activity, and chelating metal ion (Medrano-Macias et al., 2018). The greatest contents of total phenolic compounds were extracted in leaves under severe drought stress, as previously found by Nahed et al. (2019). The data indicate that total phenolic content significantly increased as MDA and drought stress levels rose. This suggests that phenolics are central to the plant’s defense mechanism against water drought -induced oxidative damage. The content of soluble or non-structural carbohydrates (NSC) also increased under drought according to Ibrahim et al. (2020). Osmotic regulation is the primary response to drought that is usually exerted by enhancing the levels of NSC and proline (Huang et al., 2019). However, proline, which is a well-known compatible osmolyte that assists plant tissues under abiotic stresses in cellular osmotic adjustment, detoxification of ROS and protection of membrane integrity and enzyme stability (Dawood and El-Awadi, 2015), decreased as drought stress increased. Indeed, its role in maintaining cellular osmotic balance and protecting cellular structures is crucial during drought. Besides, the decrease in proline levels with increased drought stress suggests plants may utilize proline reserves as a source of carbon and nitrogen when photosynthetic carbon assimilation and nitrogen uptake are restricted by drought. Thus, the reduction in proline content reflects its consumption in metabolic processes essential for the survival of the plant under extreme water deficit conditions.

The application of Fe_2_O_3_/g-C_3_N_4_ nanomaterials significantly mitigated the adverse effects of severe drought stress on olive plants. Mineral nanoparticles have great potential in modulating the effects of abiotic stresses (Zahedi et al., 2019). In fact, particularly under severe drought conditions (50% ET), the treatments boosted various pomological characteristics, such as fruit weight, length, and diameter, as well as pulp fresh weight and percentage compared to untreated controls. In addition, the Fe_2_O_3_/g-C_3_N_4_ treatment enhanced water use efficiency (WUE) in both fruit and oil production. Water Use Efficiency (WUE) is a measure of how efficiently a plant is able to use water to produce carbon through processes like photosynthesis. It can be calculated by the amount of carbon obtained (either through carbon assimilation) relative to the amount of water used or transpired by the plant. This index is pivotal to understanding the balance between water use and carbon production in plants. According to Aguilos et al. (2019), the use of carbon nanoparticles (NPs) may enhance carbon uptake, thus increasing the WUE in plants. Carbon nanomaterials are able to penetrate plant cells and enhance water uptake even under drought stress (Husen and Siddiqi, 2014). Carbon nanotubes were reported to increase the uptake efficiency of water and essential nutrients like Fe and Ca (Tiwari et al., 2014). Wang et al. (2012) demonstrated that carbon nanomaterials can enter the cell wall and cytoplasm and considerably increase the dehydrogenases’ activity of roots, thus improving the water absorption capacity of plants. Safdar et al. (2022) have shown that multi-walled carbon nanotubes can increase the permeability and flexibility of root cell membranes, promoting better water flow into the plant roots. Accordingly, the treatment improved the relative water content (RWC) while reducing the electrolyte leakage (EL), indicating better water retention and cell membrane stability under drought conditions.

In addition to enhanced water absorption and RWC, nutrient uptake (particularly Ca^2+^ and K^+^) was positively impacted by Fe_2_O_3_/g-C_3_N_4_ under drought stress, while Na^+^ accumulation was reduced. Nano-fertilizers, with their higher surface area and small size, easily penetrate plant leaves, increasing nutrient availability and use efficiency (Mahanta et al., 2019). Fe_2_O_3_/g-C_3_N_4_ treatment under severe stress resulted in the highest phenolic content and the lowest MDA levels, suggesting that this treatment was able to enhance the antioxidant capacity of the plants more effectively than the others. The superior performance of Fe_2_O_3_/g-C_3_N_4_ could be attributed to its role in bolstering the plant’s inherent defense mechanisms, particularly by enhancing the stability and effectiveness of phenolic compounds. Ghorbanpour and Hadian (2015) showed that multi-walled carbon nanotubes were able to elicit the synthesis of total phenols and flavonoids through polyphenol oxidase (PPO) activation. The activity of antioxidant enzymes like POD and CAT was also increased by Fe_2_O_3_/g-C_3_N_4_ treatment helping to manage oxidative stress caused by drought. Therefore, this treatment likely supported the maintenance of cellular integrity and function under water deficit by mitigating oxidative damage more efficiently. This lowered oxidative stress positively improved the photosynthetic pigment content (Chl a, b, and total Chl), which contributed to enhancing photosynthetic efficiency and supporting overall plant growth and productivity. Accordingly, many studies have proven that carbon NPs are able to induce antioxidant defense mechanisms (e.g., the activity of antioxidant enzymes and synthesis of antioxidant metabolites), improve water and nutrient absorption, and enhance Chl biosynthesis and stability (Chen et al., 2021; Ahmadi-Majd et al., 2022; Bakry et al., 2024). In plants exposed to drought stress, the application of NPs helps maintain stable pigment content, even though overall plant growth is slow (Babanli et al., 2024). Alabdallah et al. (2021) also reported that photosynthetic pigments decrease in plants under drought stress, and NPs may increase these pigment levels. A significant negative correlation between photosynthetic pigments and oxidative stress biomarkers was observed. The increase in pigments caused by NPs can be attributed to several factors, including enhanced carboxylation activity, lower pressure on photosynthetic electron transport chain and oxidative stress, and improved uptake of essential metals like Fe and Zn, which are involved in chlorophyll biosynthesis (Zeeshan et al., 2024).

## 5 Conclusion

Drought stress, as an increasingly widespread detrimental limiting factor, modifies numerous morphological, physiological and biochemical attributes in olive trees. Our study indicated that Fe_2_O_3_/g-C_3_N_4_ treatment has exceptional effectiveness in increasing the olive plant’s adaptability to drought stress. This remarkable enhancement is achieved through a multifaceted approach: ameliorating the uptake of water and maintaining higher relative water content (RWC), increasing the synthesis and accumulation of SNC while mobilizing proline as N reserve to satisfy the demand resulting from reduced N uptake, efficiently scavenging ROS via antioxidant enzymes and polyphenols, preserving Chls and, therefore, photosynthesis and growth. Indeed, our study offers an in-depth exploration of the regulatory effects of C_3_N_4_ nanostructures on the physiology of olive plants facing water deficiency and demonstrates that Fe_2_O_3_/g-C_3_N_4_ nanomaterials effectively enhanced the physiological resilience of olive plants under severe drought stress, leading to improved growth, yield, and stress tolerance. These findings underscore the potential of nanotechnology in agriculture and pave the way for innovative strategies against drought stress in crops. As we move forward, a critical next step will be the identification and analysis of key genes involved in drought resistance. This genetic insight will enable the development of innovative approaches to boost drought resilience and adaptation in olive plants, ensuring sustainable agriculture and productivity in semi-arid regions.

## Data Availability

The original contributions presented in the study are included in the article/supplementary material. Further inquiries can be directed to the corresponding authors.
